# Proton pump inhibitor therapy did not increase the prevalence of small-bowel injury: A propensity-matched analysis

**DOI:** 10.1371/journal.pone.0182586

**Published:** 2017-08-03

**Authors:** Atsuo Yamada, Ryota Niikura, Koutarou Maki, Masanao Nakamura, Hirotsugu Watabe, Mitsuhiro Fujishiro, Shiro Oka, Shunji Fujimori, Atsushi Nakajima, Naoki Ohmiya, Takayuki Matsumoto, Shinji Tanaka, Kazuhiko Koike, Choitsu Sakamoto

**Affiliations:** 1 Department of Gastroenterology, Graduate School of Medicine, The University of Tokyo, Tokyo, Japan; 2 Department of Gastroenterology, Nippon Medical School, Graduate School of Medicine, Tokyo, Japan; 3 Department of Gastroenterology and Hepatology, Nagoya University Graduate School of Medicine, Nagoya, Japan; 4 Wakamiya Watabe Clinic, Chiba, Japan; 5 Department of Endoscopy and Endoscopic Surgery, The University of Tokyo, Tokyo, Japan; 6 Department of Endoscopy, Hiroshima University Hospital, Hiroshima, Japan; 7 Division of Gastroenterology and Hepatology, Yokohama City University School of Medicine, Yokohama, Japan; 8 Department of Gastroenterology, Fujita Health University School of Medicine, Toyoake, Japan; 9 Division of Gastroenterology, Department of Internal Medicine, Iwate Medical University, Morioka, Japan; University Hospital Llandough, UNITED KINGDOM

## Abstract

**Background:**

Previous studies have reported that the suppression of acid secretion by using proton pump inhibitors (PPIs) results in dysbiosis of the small-bowel microbiota, leading to exacerbated small-bowel injuries, including erosions and ulcers. This study was designed to assess the association between PPI therapy and small-bowel lesions after adjustment for the differences in baseline characteristics between users and non-users of PPIs.

**Methods:**

We retrospectively studied patients suspected to be suffering from small-bowel diseases, who underwent capsule endoscopy between 2010 and 2013. We used propensity matching to adjust for the differences in baseline characteristics between users and non-users of PPIs. The outcomes included the prevalence of small-bowel lesions: erosion, ulcer, angioectasia, varices, and tumor.

**Results:**

We selected 327 patient pairs for analysis after propensity matching, and found no significant differences in the prevalence of small-bowel injuries, including erosions and ulcers, between users and non-users of PPIs. Two subgroup analyses of the effect of the type of PPI and the effect of PPI therapy in users and non-users of nonsteroidal anti-inflammatory drugs indicated no significant differences in the prevalence of small-bowel injuries in these two groups.

**Conclusion:**

PPI therapy did not increase the prevalence of small-bowel injury, regardless of the type of PPI used and the use of nonsteroidal anti-inflammatory drugs.

## Introduction

Proton pump inhibitors (PPIs) have been widely used in the treatment of gastroesophageal reflux disease, peptic ulcer, and gastrointestinal (GI) injuries associated with the use of nonsteroidal anti-inflammatory drugs (NSAIDs) and aspirin[1–3]. However, the protective and adverse effects of PPIs on the small intestine remain unknown.

In an experimental study, Wallace et al. suggested that the suppression of acid secretion using PPIs resulted in dysbiosis of the microbiome of the small bowel and exacerbated NSAID-induced enteropathy[4, 5]. Few clinical studies have evaluated the effect of PPI therapy on the small-bowel mucosa. Most studies have focused on the evaluation of highly selected patients such as those who use NSAIDs and low-dose aspirin (LDA) [6–9]. No previous studies have evaluated whether PPI therapy affects the small-bowel mucosa in non-users of NSAIDs.

The risk of small-bowel lesions has been reported to be dependent on drug therapy and comorbidities[9–13]. To assess the association between PPI therapy and small-bowel lesions, differences in baseline characteristics between users and non-users of PPIs need to be adjusted. In this regard, propensity-score matching is used to reduce the selection bias and potential confounders, and to construct a randomized controlled trial-like model in which the observed outcomes in the intervention groups can be compared[14]. In the present study, we conducted a propensity-matched analysis to assess the association between PPI therapy and small-bowel lesions by using a large multicenter capsule endoscopy database.

## Methods

### Study design, setting, and participants

We used a prospective capsule endoscopy database originally designed by the Japanese Association for Capsule Endoscopy (JACE). This database prospectively registered consecutive patients who underwent capsule endoscopy (CE) at 16 referral centers and two regional centers in Japan between November 2010 and August 2013. All CE procedures were performed using PillCam^®^ SB2 capsule endoscopy (Covidien, Dublin, Ireland). Endoscopists with experience in CE at each institution independently assessed the CE images and discussed their findings with other endoscopists, and a consensus was reached in each hospital. Each endoscopist inputted patient data and endoscopic findings in this database immediately after the CE procedure. This database also included data on the purpose of CE, and detailed patient information, including comorbidities, drug therapies, and laboratory data. CE in patients registered in the database was indicated for the diagnosis of 1) obscure gastrointestinal bleeding[15], 2) small-bowel tumors, 3) gastrointestinal symptoms, including recurrent abdominal pain and diarrhea, and 4) inflammatory bowel disease. The database included data from 1,769 patients who underwent CE. We systematically excluded 33 patients with insufficient data and 101 patients with a previous diagnosis of small-bowel ulcer/erosion or angioectasia. Therefore, a total of 1635 patients who underwent CE were considered eligible. This study complied with the Declaration of Helsinki and was approved by the Research Ethics Committee of The University of Tokyo, Nippon Medical School and other related institutions. This study was a retrospective study, not an intervention study to human subjects, and the data were analyzed anonymously. Therefore, patient informed consent to participate was not required.

### Variables and outcomes

We evaluated patient characteristics including age, sex, smoking, alcohol consumption, comorbidities, drug therapies, and CE findings. The comorbidities evaluated were hypertension, dyslipidemia, diabetes mellitus, ischemic heart disease (including history of myocardial infarction or angina pectoris), valvular disease of the mitral and aortic valves, chronic heart failure, chronic renal disease, peptic ulcer, Crohn’s disease, ulcerative colitis, collagen disease, liver cirrhosis, cancer, lymphoma, and leukemia. Drug therapy was evaluated for the following medications: NSAIDs, LDA, thienopyridine, dipyridamole, icosapentate, beraprost, sarpogrelate, limaprost, warfarin, non-vitamin K antagonist oral anticoagulants, steroids, pregabalin, PPIs, histamine H2-receptor antagonists, and mucosal protective agents. Drug therapy was defined as oral administration starting at least one month before CE.

The primary outcome was the prevalence of significant small-bowel lesions. Significant lesions were categorized as follows[10, 13, 16, 17]: erosion or ulcer—a central pallor and surrounding erythema and loss of villi; angioectasia—a circumscribed patchy, flat, sharply demarcated reddened area; small-bowel varices—distended, tortuous, or saccular veins; and tumor—a protruded lesion with mucosal change.

### Statistical analysis

**Fig 1:**
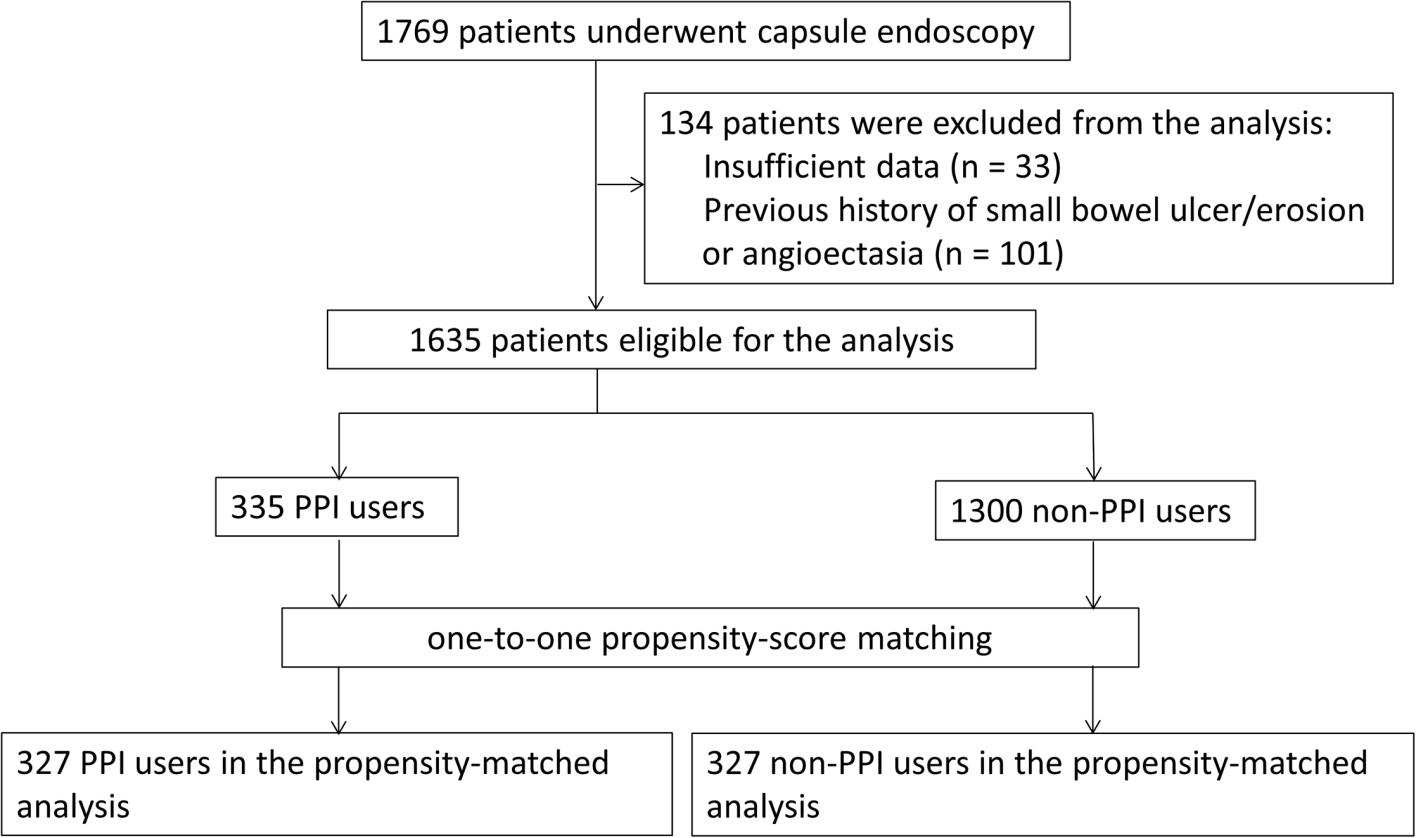
Study flow chart. PPI, proton pump inhibitor.

The propensity score was estimated using a logistic regression model for PPI users as a function of patient demographic and drug therapy data. We included 34 factors that were considered potentially clinically significant variables: age; sex; smoking; drinking; all comorbidities; and the use of medications including NSAIDs, LDA, thienopyridine, dipyridamole, icosapentate, beraprost, sarpogrelate, limaprost, warfarin, non-vitamin K antagonist oral anticoagulants, steroids, pregabalin, and mucosal protective agents. Some of these variables significantly differed between users and non-users of PPIs. We performed a one-to-one matching analysis between users and non-users of PPIs, using the nearest neighbor method within a caliper of width of 0.2 of the standard deviation of the logit of the propensity score (Fig 1). After propensity matching, the differences in the prevalence of significant small-bowel lesions were compared between the two groups. Two subgroup analyses of the effect of the type of PPI and the effect of PPI therapy in users and non-users of NSAIDs were also performed. To estimate the influence of protopathic bias, we performed a subgroup analysis of patients who could be predictable indications for PPI therapy, such as patients with a previous history of peptic ulcer, and those with a history of NSAID or aspirin use, using another propensity-matched analysis. P-values < 0.05 were considered statistically significant. All statistical analyses were conducted using STATA^®^ software version 13 (Stata Corporation, College Station, TX, USA) and JMP^®^ Pro software version 11 (SAS Institute Inc., Cary, NC, USA).

## Results

### Patient selection

The demographic and clinical characteristics of the patients (n = 1635) and propensity score-matched patients (n = 654) are shown in Table 1. Some differences were found between the groups before propensity score matching. The group subjected to PPI therapy consisted of a higher proportion of older and male patients, and patients with the highest rate of comorbidities and use of NSAIDs, antiplatelet or anticoagulant drugs, and steroids. The area under the receiver operating characteristic curve for propensity scores for PPI use was 0.737 (95% confidence interval, 0.699–0.786). After one-to-one propensity score matching, 327 pairs of users and non-users of PPIs were selected, and both groups had similar characteristics.

**Table 1:** Demographic and clinical characteristics of users and non-users of proton pump inhibitors.

	All patients					Propensity-matched patients				
	PPI (+)		PPI (–)			PPI (+)		PPI (–)		
	(n = 335)		(n = 1300)			(n = 327)		(n = 327)		
Characteristics	n	%	n	%	P	n	%	n	%	P
Age ≥65 y	201	(60.0)	642	(49.4)	<0.01	196	(59.9)	207	(63.3)	0.38
Sex (male)	200	(59.7)	859	(66.1)	0.029	196	(29.9)	200	(61.2)	0.75
Hemoglobin concentration <11 g/dL^†^	60	(43.8)	174	(51.3)	0.14	57	(42.9)	56	(46.7)	0.54
Habit										
Drinking	97	(29.0)	288	(22.2)	<0.01	92	(28.1)	78	(23.9)	0.21
Smoking	86	(25.7)	182	(14.0)	<0.01	81	(24.8)	72	(22.0)	0.41
Comorbidity										
Hypertension	55	(16.4)	146	(11.2)	0.01	53	(16.2)	55	(16.8)	0.83
Diabetes mellitus	77	(23.0)	192	(14.8)	<0.01	73	(22.3)	76	(23.2)	0.78
Hyperlipidemia	63	(18.8)	151	(11.6)	<0.01	60	(18.4)	48	(14.7)	0.21
Ischemic heart disease	58	(17.3)	136	(10.5)	<0.01	55	(16.8)	51	(15.6)	0.67
Valvular disease	41	(12.2)	68	(5.2)	<0.01	39	(11.9)	39	(11.9)	1.0
Chronic heart failure	20	(6.0)	44	(3.4)	0.03	19	(5.8)	19	(5.8)	1.0
Chronic renal failure	53	(15.8)	132	(10.2)	<0.01	50	(15.3)	60	(18.4)	0.30
Peptic ulcer	29	(8.7)	107	(8.2)	0.80	29	(8.9)	30	(9.2)	0.89
Crohn’s disease	7	(2.1)	60	(4.6)	0.038	7	(2.1)	13	(4.0)	0.26
Ulcerative colitis	5	(1.5)	20	(1.5)	0.95	5	(1.5)	3	(0.9)	0.73
Collagen disease	13	(3.9)	27	(2.1)	0.057	12	(3.7)	11	(3.4)	1.0
Liver cirrhosis	65	(19.4)	118	(9.1)	<0.01	61	(18.7)	73	(22.3)	0.25
Cancer	23	(6.9)	40	(3.1)	<0.01	22	(6.7)	29	(8.9)	0.31
Lymphoma	7	(2.1)	39	(3.0)	0.46	7	(2.1)	6	(1.8)	1.0
Leukemia	7	(2.1)	12	(0.9)	0.087	7	(2.1)	9	(2.8)	0.80
Medication										
NSAIDs	32	(9.6)	76	(5.9)	0.015	29	(8.9)	30	(9.2)	0.89
LDA	109	(32.5)	185	(14.2)	<0.01	104	(31.8)	103	(31.5)	0.93
Thienopyridine	38	(11.3)	46	(3.5)	<0.01	35	(10.7)	34	(10.4)	0.90
Dipyridamole	1	(0.3)	3	(0.2)	1.0	1	(0.31)	3	(0.92)	0.62
Icosapentate	5	(1.5)	9	(0.7)	0.18	5	(1.5)	6	(1.8)	1.0
Beraprost	0	(0)	1	(0.1)	1.0	0	(0)	0	(0)	N.A.
Sarpogrelate	2	(0.6)	2	(0.2)	0.19	1	(0.31)	1	(0.31)	1.0
Limaprost	4	(1.2)	11	(0.9)	0.53	4	(1.2)	2	(0.61)	0.69
Warfarin	52	(15.5)	71	(5.5)	<0.01	48	(14.7)	47	(14.4)	0.91
NOACs	2	(0.6)	5	(0.4)	0.64	2	(0.61)	2	(0.61)	1.0
Steroids	36	(10.8)	30	(2.3)	<0.01	33	(10.1)	29	(8.9)	0.69
Pregabalin	5	(1.5)	3	(0.2)	0.011	4	(1.2)	2	(0.6)	0.69
Mucosal protection agents	79	(23.6)	145	(11.2)	<0.01	77	(23.6)	76	(23.2)	1.0

PPI, proton pump inhibitor

NSAID, non-steroidal anti-inflammatory drug

LDA, low-dose aspirin

NOAC, non-vitamin K antagonist oral anticoagulant

N.A., not applicable

†Factors included missing data.

### Significant small-bowel lesions

Significant lesions in each group of the propensity-matched patients are shown in Table 2. No significant differences in the prevalence of erosion or ulcer, angioectasia, varices, and tumor were found between the groups.

**Table 2:** Association between proton pump inhibitor therapy and significant small-bowel lesions in propensity-matched patients.

	PPI (+)	PPI (–)	Crude odds ratio	P
	(n = 327)	(n = 327)		
	n (%)	n (%)	(95% CI)	
Erosion/ulcer	93 (28.4)	85 (26.0)	1.1 (0.85–1.4)	0.48
Angioectasia	36 (11.0)	26 (8.0)	1.4 (0.86–2.2)	0.18
Varix	3 (0.92)	1 (0.31)	3.0 (0.31–29)	0.31
Tumor	18 (5.5)	21 (6.4)	0.86 (0.47–1.6)	0.62

PPI, proton pump inhibitor

CI, confidence interval

Significant small-bowel lesions in each group in the subgroup analysis of the type of PPIs used are shown in Table 3. No significant differences in the prevalence of erosion or ulcer, angioectasia, varices, and tumor were found when different PPIs were used.

**Table 3:** Subgroup analysis of the association between the type of PPI used and significant small-bowel lesions.

	PPI (+)	PPI (−)	Crude odds ratio	P
	n (%)	n (%)	(95% CI)	
Lansoprazole	121	121		
Erosion/ulcer	44 (36)	32 (26)	1.4 (0.94–2.0)	0.09
Angioectasia	16 (13)	10 (8)	1.6 (0.76–3.4)	0.21
Varices	0 (0)	0 (0)	N.A.	N.A.
Tumor	6 (5.0)	6 (5.0)	1.0 (0.3–3.0)	1.0
Omeprazole	67	67		
Erosion/ulcer	14 (20)	14 (20)	1.0 (0.52–1.9)	1.0
Angioectasia	5 (7.5)	8 (7.5)	0.63 (0.22–1.8)	0.39
Varices	1 (1.5)	0 (0)	N.A.	N.A.
Tumor	2 (3)	5 (7)	0.4 (0.08–2.0)	0.26
Rabeprazole	139	139		
Erosion/ulcer	35 (25)	39 (28)	0.90 (0.61–1.3)	0.59
Angioectasia	15 (11)	8(6)	1.9 (0.82–4.3)	0.13
Varices	2 (1.4)	1 (0.7)	2.0 (0.18–22)	0.56
Tumor	10 (7)	10 (7)	1.0 (0.43–2.3)	1.0

PPI, proton pump inhibitor

CI, confidence interval

N.A., not applicable

Significant lesions in each group in the subgroup analysis of the effect of PPI therapy in users and non-users of NSAIDs are shown in Table 4. Among the NSAID users, no significant differences in the prevalence of erosion or ulcer, angioectasia, varices, and tumor were found between the groups. Similarly, PPI therapy caused no significant differences in the prevalence of small-bowel lesions among non-users of NSAIDs.

**Table 4:** Subgroup analysis of the association between PPI therapy and significant small-bowel lesions in users and non-users of nonsteroidal anti-inflammatory drugs.

	PPI (+)	PPI (−)	Crude odds ratio	P
	n (%)	n (%)	(95% CI)	
Users of NSAIDs	29	30		
Erosion/ulcer	14 (48)	19 (63)	0.76 (0.48–1.2)	0.24
Angioectasia	4 (14)	1 (3)	4.1 (0.49–35)	0.14
Varices	0 (0)	0 (0)	N.A.	N.A.
Tumor	1 (3)	1 (3)	1.0 (0.07–16)	0.98
Non-users of NSAIDs	298	297		
Erosion/ ulcer	79 (27)	66 (22)	1.2 (0.90–1.6)	0.22
Angioectasia	32 (11)	25 (8)	1.3 (0.78–2.1)	0.31
Varices	3 (1)	1 (0.3)	3.0 (0.31–29)	0.34
Tumor	17 (6)	20 (7)	0.85 (0.45–1.6)	0.60

PPI, proton pump inhibitor

NSAIDs, nonsteroidal anti-inflammatory drugs

CI, confidence interval

N.A., not applicable

To estimate the influence of protopathic bias, we performed a subgroup analysis of patients who could be predictable indications for PPI therapy, such as patients with a previous history of peptic ulcer, and those with a history of NSAID or aspirin use, using another propensity-matched analysis (S1 and S2 Tables). In this subgroup analysis, no significant differences in the prevalence of erosion or ulcer, angioectasia, varices, and tumor were found between the groups.

## Discussion

We evaluated the association between PPI therapy and significant small-bowel lesions using a large CE database, and indicated that the prevalence of small-bowel injuries including erosion and ulcers did not increase with PPI therapy, regardless of the type of PPI used. This association also existed in non-users as well as users of NSAIDs.

Gastric acid prevents bacterial colonization of the upper gastrointestinal tract and can influence the proper composition of the intestinal flora. The suppression of acid secretion with PPIs may lead to bacterial overgrowth in the stomach and small intestine[5]. In an experimental study, Wallace et al. demonstrated that the use of PPIs had a minimum detectable effect on the small-bowel mucosa, although treatment with a PPI alone caused significant changes in the small-bowel microbiome[4]. However, to the best of our knowledge, no clinical studies have evaluated the effect of PPIs on the small-bowel mucosa. Our results revealed that the use of PPIs did not increase the prevalence of small-bowel injuries, regardless of the type of PPI used. It is believed that the alterations in the lumen due to PPIs do not lead to small-bowel injuries.

Our study also showed that PPIs combined with NSAIDs did not increase the risk of small-bowel injuries, which is inconsistent with the results of several studies[7–9]. Watanabe et al. evaluated small-bowel mucosal injuries in NSAID users using CE, and found that PPI therapy was an independent risk factor for the development of severe NSAID-induced small-bowel damage[7]. Washio et al. conducted a prospective randomized controlled trial and demonstrated that the incidence of small-bowel injuries was higher in subjects treated with celecoxib plus rabeprazole than in those treated with celecoxib alone[8]. On the contrary, Ishihara et al. evaluated the risk factors for symptomatic NSAID-induced small bowel injuries and revealed that PPI did not increase the risk of symptomatic NSAID-induced small bowel injury[18]. In an experimental study, Wallace et al. found that PPIs exacerbated NSAID-induced small-bowel mucosal injuries via PPI-induced dysbiosis[4]. However, other studies have reported contradictory results on the association between small-bowel injuries and PPI therapy[19, 20]. The evidence on this association is limited; therefore, further studies on other populations are required to evaluate the association between the combined use of PPIs and NSAIDs, and small-bowel injuries.

This study has some strengths. First, we assessed a large number of patients in a multicenter study, and all subjects underwent CE. Second, the prospective collection of baseline characteristics, including drugs and comorbidities, was thorough. However, the study also has some limitations. First, information on the duration of and indication for PPI therapy was not collected. Accordingly, protopathic bias may influence the study outcome. In a subgroup analysis of patients who could be predictable indications for PPI therapy, such as patients with a previous history of peptic ulcer, or those with a history of NSAID or aspirin use, no effect of PPIs was observed on small-bowel lesions. Therefore, we speculated that the lack of data on PPIs had a limited influence on our conclusion. Second, although we used a propensity-matched analysis in order to reduce the bias in causal estimates owing to observed differences between the treatment groups, our study is still subject to biases from unobserved differences. Unmeasurable confounders associated with this therapy, including clinical indication, underlying diseases, and medications, may exist. Third, all patients who underwent CE were suspected to be suffering from small-bowel diseases; therefore, our results could not be extended to all PPI users.

In conclusion, the prevalence of small-bowel injuries did not increase with PPI therapy, regardless of the type of PPI used and the use of NSAIDs.

## Supporting information

S1 Table Demographic and clinical characteristics of users and non-users of proton pump inhibitors in the subgroup of patients with previous diagnosis of peptic ulcer and NSAIDs or aspirin users. PPI, proton pump inhibitor. NSAID, non-steroidal anti-inflammatory drug. LDA, low-dose aspirin. NOAC, non-vitamin K antagonist oral anticoagulants. N.A., not applicable. ^†^Factors included missing data.
(DOCX)

S2 Table Association between proton pump inhibitor therapy and significant small-bowel lesions in the subgroup of patients with previous diagnosis of peptic ulcer, and NSAID or aspirin users. CI, confidence interval. PPI, proton pump inhibitor.
(DOCX)
