# Supplementary material for: Proton pump inhibitor therapy did not increase the prevalence of small-bowel injury: A propensity-matched analysis
Source: PLoS One. 2017 Aug 3;12(8):e0182586. doi: 10.1371/journal.pone.0182586 (PMC5542471; doi:10.1371/journal.pone.0182586)
Supplement: S1 Table — PPI, proton pump inhibitor. NSAID, non-steroidal anti-inflammatory drug. LDA, low-dose aspirin. NOAC, non-vitamin K antagonist oral anticoagulants. N.A., not applicable. †Factors included missing data. (DOCX) [file pone.0182586.s001.docx]

**S1 Table. Demographic and clinical characteristics of users and non-users of proton pump inhibitors in the subgroup of patients with previous diagnosis of peptic ulcer and NSAIDs or aspirin users**

|  | **All patients** | | | | | **Propensity-matched patients** | | | | |
| --- | --- | --- | --- | --- | --- | --- | --- | --- | --- | --- |
|  | PPI (+) | | PPI (–) | |  | PPI (+) | | PPI (–) | |  |
|  | (n = 154) | | (n = 341) | |  | (n = 105) | | (n = 105) | |  |
| Characteristics | n | (%) | n | (%) | P | n | (%) | n | (%) | P |
| Age ≥65 y | 99 | (64.3) | 239 | (70.1) | 0.20 | 70 | (66.7) | 66 | (62.9) | 0.56 |
| Sex (male) | 94 | (61.0) | 235 | (68.9) | 0.086 | 65 | (61.9) | 67 | (63.8) | 0.78 |
| Hemoglobin concentration <11 g/dL^†^ | 28 | (43.1) | 72 | (48.3) | 0.48 | 21 | (43.8) | 24 | (50.0) | 0.54 |
| Habit |  |  |  |  |  |  |  |  |  |  |
| Drinking | 44 | (28.6) | 79 | (23.2) | 0.20 | 28 | (26.7) | 28 | (26.7) | 1.0 |
| Smoking | 50 | (32.5) | 62 | (18.2) | <0.01 | 29 | (27.6) | 31 | (29.5) | 0.88 |
| Comorbidity |  |  |  |  |  |  |  |  |  |  |
| Hypertension | 27 | (17.5) | 72 | (21.1) | 0.36 | 18 | (17.1) | 18 | (17.1) | 1.0 |
| Diabetes mellitus | 43 | (27.9) | 83 | (24.3) | 0.40 | 30 | (28.6) | 25 | (23.8) | 0.43 |
| Hyperlipidemia | 5 | (3.2) | 10 | (2.9) | 0.85 | 25 | (23.8) | 22 | (21.0) | 0.62 |
| Ischemic heart disease | 59 | (38.3) | 106 | (31.1) | 0.11 | 38 | (36.2) | 29 | (27.6) | 0.18 |
| Valvular disease | 22 | (14.3) | 29 | (8.5) | 0.05 | 15 | (14.3) | 15 | (14.3) | 1.0 |
| Chronic heart failure | 13 | (8.4) | 14 | (4.1) | 0.049 | 10 | (9.5) | 8 | (7.6) | 0.81 |
| Chronic renal failure | 29 | (18.8) | 58 | (17.0) | 0.62 | 22 | (21.0) | 18 | (17.1) | 0.48 |
| Peptic ulcer | 32 | (20.8) | 110 | (32.3) | <0.01 | 25 | (23.8) | 26 | (24.8) | 0.87 |
| Crohn’s disease | 0 | (0) | 2 | (0.59) | 0.53 | 0 | (0) | 0 | (0) | N.A. |
| Ulcerative colitis | 1 | (0.65) | 1 | (0.29) | 0.53 | 1 | (1) | 0 | (0) | N.A. |
| Collagen disease | 5 | (3.3) | 10 | (2.9) | 0.79 | 1 | (1) | 3 | (2.9) | 0.2 |
| Liver cirrhosis | 11 | (7.1) | 26 | (7.6) | 0.85 | 7 | (6.7) | 10 | (9.5) | 0.61 |
| Cancer | 5 | (3.3) | 15 | (4.4) | 0.63 | 3 | (2.9) | 4 | (3.8) | 1.0 |
| Lymphoma | 0 | (0) | 3 | (0.88) | 0.56 | 0 | (0) | 3 | (2.9) | 0.25 |
| Leukemia | 1 | (0.65) | 4 | (1.2) | 1.0 | 1 | (1) | 1 | (1) | 1.0 |
| Medication |  |  |  |  |  |  |  |  |  |  |
| NSAIDs | 35 | (22.7) | 83 | (24.3) | 0.70 | 26 | (24.8) | 24 | (22.9) | 0.75 |
| LDA | 112 | (72.7) | 196 | (57.5) | <0.01 | 74 | (70.5) | 71 | (67.6) | 0.65 |
| Thienopyridine | 34 | (22.1) | 30 | (8.8) | <0.01 | 18 | (17.1) | 14 | (13.3) | 0.44 |
| Dipyridamole | 0 | (0) | 2 | (0.6) | 1.0 | 0 | (0) | 0 | (0) | N.A. |
| Icosapentate | 3 | (2.0) | 4 | (1.2) | 0.68 | 3 | (2.9) | 0 | (0) | 0.25 |
| Beraprost | 0 | (0) | 1 | (0.3) | 1.0 | 0 | (0) | 0 | (0) | N.A. |
| Sarpogrelate | 1 | (0.7) | 2 | (0.6) | 1.0 | 0 | (0) | 0 | (0) | N.A. |
| Limaprost | 5 | (3.3) | 6 | (1.8) | 0.33 | 1 | (1) | 3 | (2.9) | 0.62 |
| Warfarin | 30 | (19.5) | 34 | (10.0) | <0.01 | 18 | (17.1) | 20 | (19.1) | 0.72 |
| NOACs | 1 | (0.7) | 1 | (0.3) | 0.53 | 0 | (0) | 0 | (0) | N.A. |
| Steroids | 15 | (9.7) | 11 | (3.2) | <0.01 | 6 | (5.7) | 7 | (6.7) | 1.0 |
| Pregabalin | 3 | (2.0) | 2 | (0.6) | 0.18 | 2 | (2) | 0 | (0) | 0.50 |
| Mucosal protection agents | 45 | (29.2) | 56 | (16.4) | <0.01 | 20 | (19.1) | 18 | (17.1) | 0.72 |

PPI, proton pump inhibitor

NSAID, non-steroidal anti-inflammatory drug

LDA, low-dose aspirin

NOAC, non-vitamin K antagonist oral anticoagulants

N.A., not applicable

^†^Factors included missing data.
