# Supplementary material for: Proton pump inhibitor therapy did not increase the prevalence of small-bowel injury: A propensity-matched analysis
Source: PLoS One. 2017 Aug 3;12(8):e0182586. doi: 10.1371/journal.pone.0182586 (PMC5542471; doi:10.1371/journal.pone.0182586)
Supplement: S2 Table — CI, confidence interval. PPI, proton pump inhibitor. (DOCX) [file pone.0182586.s002.docx]

**S2 Table. Association between proton pump inhibitor therapy and significant small-bowel lesions in the subgroup of patients with previous diagnosis of peptic ulcer, and NSAID or aspirin users**

|  | PPI (+) | PPI (−) | Crude odds ratio | P |
| --- | --- | --- | --- | --- |
|  | (n = 105) | (n = 105) |  |  |
|  | n (%) | n (%) | (95% CI) |  |
| Erosion/ulcer | 36 (34.3) | 32 (30.5) | 1.2 (0.62–2.2) | 0.66 |
| Angioectasia | 10 (9.5) | 11 (10.5) | 0.90 (0.36–2.2) | 0.82 |
| Varix | 0 | 0 | N.A. | N.A. |
| Tumor | 2 (1.9) | 1 (1) | 2.0 (0.18–21) | 1.0 |

CI, confidence interval

PPI, proton pump inhibitor
